# Molecular docking and MD simulation approach to identify potential phytochemical lead molecule against triple negative breast cancer

**DOI:** 10.12688/f1000research.155657.1

**Published:** 2024-10-24

**Authors:** Pranaya Sankaranarayanan, Dicky John Davis G, Abhinand PA, M Manikandan, Arabinda Ghosh

**Affiliations:** 1 Department of Bioinformatics, Sri Ramachandra Institute of Higher Education and Research (Deemed to be University), Chennai, Tamil Nadu, 600116, India; 2 Department of Medical Genetics, Manipal Hospitals, Bengaluru, Karnataka, 560 017, India; 3 Department of Botany, Gauhati University, Guwahati, Assam, India

**Keywords:** Triple Negative Breast Cancer, AR target, Phytochemicals, 2–hydroxy naringenin, Virtual screening, Molecular Docking, Molecular dynamics simulation.

## Abstract

**Background:**

Triple-negative breast cancers are defined as tumors that lack the expression of the estrogen receptor (ER), progesterone receptor (PR), and human epidermal growth factor receptor 2 (HER2). It exhibits unique clinical and pathological features, is highly aggressive, and has a relatively poor prognosis and poor clinical outcome.

**Objective:**

To identify a novel drug target protein against triple-negative breast cancer (TNBC) and potential phytochemical lead molecules against novel drug targets.

**Methods:**

In this study, we retrieved TNBC samples from NGS and microarray datasets in the Gene Expression Omnibus database and employed a combination of differential gene expression studies, protein-protein interaction analysis, and network topology investigation to identify the target protein. Using molecular docking and molecular dynamics simulation studies, followed by Molecular Mechanics with Generalised Born Surface Area solvation, a potential lead molecule was identified.

**Result:**

The androgen receptor (AR) was found to be the target protein, and 2-hydroxynaringenin was discovered to be a possible phytochemical lead molecule to combat TNBC.

Upregulated genes with LogFC > 1.25 and P-value < 0.05 from the TNBC gene expression dataset were given to STRING tool to investigate the network topology, and androgen receptor (AR) was found to be an appropriate hub gene in the protein-protein interaction network. Phytochemicals that inhibit breast cancer were retrieved from the PubChem database and virtual screening was performed using PyRx against the AR protein. Based on Lipinski’s rule and ADMET properties, molecular interaction studies were analyzed using induced fit docking, wherein significant binding interactions were displayed by 2-hydroxynaringenin. Molecular dynamics studies and MM-GBSA of AR and the 2-hydroxynaringenin complex revealed strong and stable interactions.

**Conclusion:**

AR was identified as a hub protein that is highly expressed in breast cancer and 2-hydroxynaringenin efficacy of counter TNBC needs to be investigated further in vitro and in vivo.

**Abbreviations**
ADMET: Absorption, Distribution, Metabolism, Excretion and Toxicity
AR: Androgen Receptor
DEG: Differentially Expressed Genes
ER: Estrogen Receptor
GEO: Gene Expression Omnibus
HER2: Human Epidermal Growth Factor Receptor 2
MCODE: Molecular Complex Detection
MD: Molecular Dynamics
MM-GBSA: Molecular Mechanics with Generalized Born and Surface Area Solvation
NCBI: National Center for Biotechnology Information
pcR: Pathological Complete Response
PDB: Protein Data Bank
PPI: Protein–Protein Interactions
PR: Progesterone Receptor
TNBC: Triple Negative Breast Cancer

## Introduction

Breast cancer is the most common type of cancer worldwide, as reported by the World Health Organization (WHO) in 2020 with over 7.8 million women living in the last five years diagnosed with breast cancer. ^1^ It is responsible for 685,000 deaths worldwide. However, it should be noted that breast cancer is a non-homogenous condition that can be classified into several significant subtypes based on the expression of their genes. Triple-negative breast cancers (TNBC) are characterized by the absence of estrogen, progesterone, and ERBB2 receptors, and are specifically identified as estrogen receptor (ER)-negative, progesterone receptor (PR)-negative, and human epidermal growth factor receptor 2 (HER2). TNBC accounts for 12%–17% of all breast cancers. ^2^ Sandhu et al. revealed a considerably greater prevalence of TNBC in India than in Western populations. Approximately one in three women diagnosed with breast cancer in India was found to have triple-negative disease.

Triple-Negative Breast Cancer exhibits unique clinical and pathologic features, is highly aggressive, and has a relatively poor prognosis and clinical outcome. ^3^ Currently, there is no recognized targeted treatment for TNBC. The primary treatment options for TNBC involve chemotherapy utilizing anthracyclines, taxanes, and/or platinum compounds as the major treatment modalities. A significant proportion of TNBC patients fail to attain Pathological Complete Response (pCR) with standard chemotherapy, prompting concerns about the effectiveness and safety of the chosen chemotherapy. ^4^ A better understanding of the pathological mechanisms of TNBC onset and progression and the molecular interactions underlying the etiology of the condition can help improve the prophylaxis and design of novel targeted treatment against this cancer type. ^5^

Gene expression profiling can be invaluable for detecting transcriptional variations between normal and malignant cells and can be extensively used to study gene phenotype associations in breast neoplasms. ^6^ Protein interaction networks potentially signify patterns in network connectivity between proteins, which can differ between breast cancer subtypes. ^7^ Phytochemicals are natural, non-toxic compounds found in plants that possess disease-protective or preventive properties. ^8^ They modulate the molecular pathways associated with cancer growth and progression. ^9^

The present study aimed to identify a novel therapeutic target protein for TNBC by integrating differential gene expression studies with protein-protein interactions and network topology analysis. Subsequently, phytochemicals with reported anti-breast cancer activities will be subjected to virtual screening by molecular docking against the identified novel target. To validate these findings, Molecular Mechanics with Generalised Born Surface Area solvation and Molecular Dynamics simulations were performed. Based on their binding affinity to the target protein, novel therapeutic phytochemical lead molecules with anti-TNBC activity were identified.

## Method

### Gene expression profiling of TNBC microarray datasets

**Figure 1.**
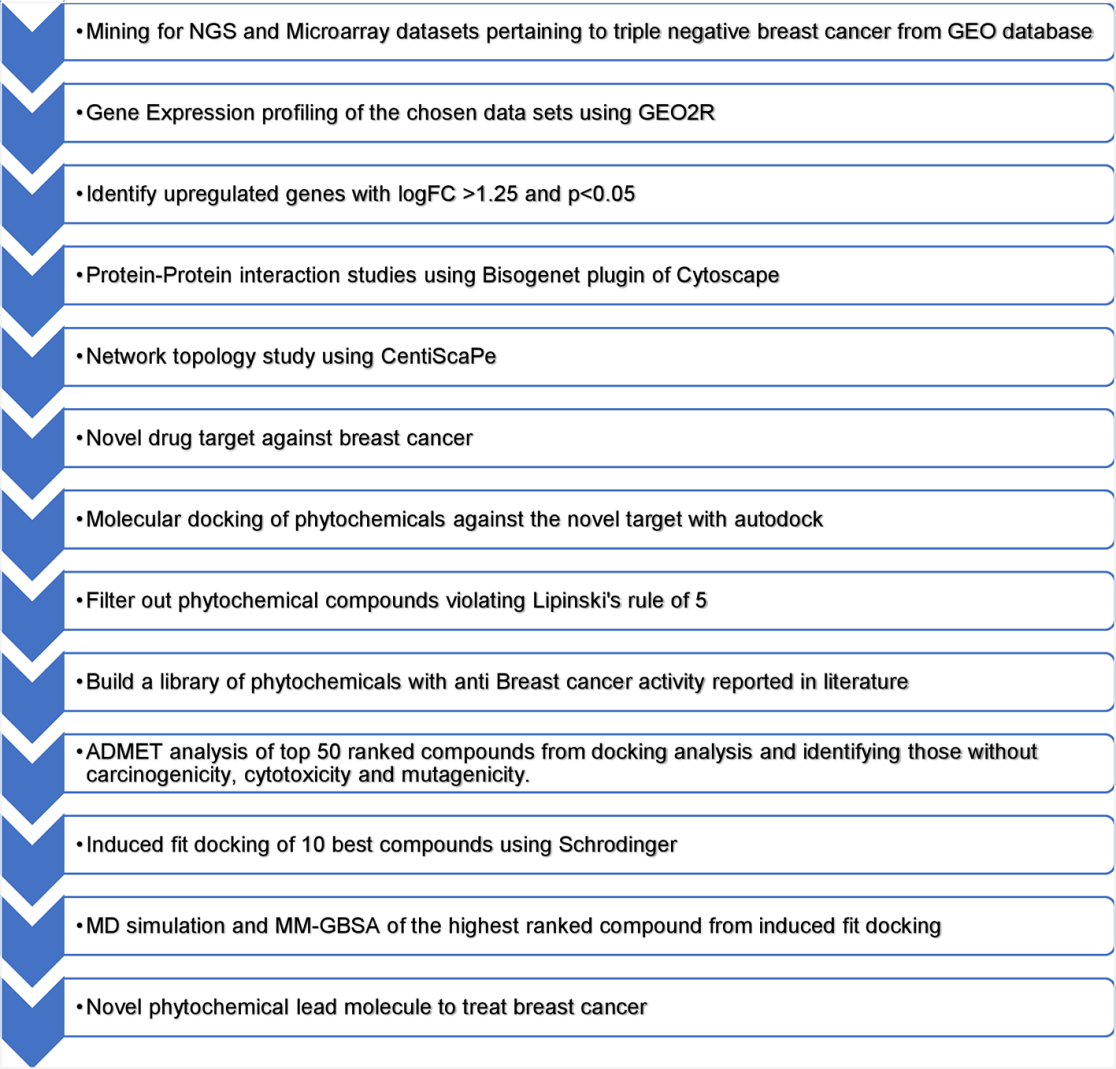
Overview of methodology.

A thorough literature mining effort encompassing all eligible studies on gene expression in TNBC was conducted. The search involved querying the Gene Expression Omnibus (GEO) datasets. Gene expression profiling was performed using GEO2R to identify significantly upregulated genes. Figure 1 presents an overview of the methodology.

During the literature mining process, a microarray dataset was obtained from the NCBI GEO repository using the accession number GSE45498 annotated in the GPL16299 platform. This dataset encompasses 40 samples from healthy normal tissues, 160 from individuals with cancer, and 54 from metastatic cases. NGS datasets were obtained from the NCBI GEO repository using accession number GSE214101 annotated in the GPL20301 platform. This dataset included 24 samples derived from the MDA-MB-231 and MDA-MB-436 cell lines. Gene expression profiling values underwent log (base2) transformation and percentage shift normalization was applied. To assess the differences in gene expression between normal and diseased samples, the fold change for each gene was individually calculated. A threshold of 1.25-fold change was used to categorize genes as being upregulated. Gene expression profiling followed the protocol reported previously. ^10^

### Study of protein–protein interactions

The selected genes were subjected to the Bisogenet plug-in of Cytoscape to identify protein-protein interactions of all genes differentially regulated in TNBC. STRING is an open-source bioinformatics platform integrated in Cytoscape, designed for the study of both predicted and known protein-protein interactions. This database gathers, evaluates, and integrates information on protein-protein interactions from all publicly available sources. Additionally, it augments these data with computational predictions. ^11^ These interactions encompass both indirect (functional) and direct (physical) associations. ^12^ The genes were uploaded and a string network was built. Molecular Complex Detection (MCODE) detects Protein-Protein Interactions subnetworks and highly interconnected clusters within the PPI network. ^13^ PPI networks were broken down into top-ranked dense cliques (sub-clusters) using the MCODE plugin. The top-ranked dense clique was selected for further analysis.

### Building a library of phytochemicals with anti- breast cancer activity

Phytochemicals are naturally occurring biologically active chemical compounds found in plants that serve as medicinal ingredients and nutrients, offering health benefits to humans. ^14^ Many natural products and their analogs have been identified as potent anticancer agents and the anticancer properties of various plants and phytochemicals. ^15^ Phytochemicals were identified through a systematic literature search indicating anti-breast cancer activity were selected, and their 3D structures in SDF format were retrieved from PubChem database. Subsequently, phytochemicals that did not conform to Lipinski’s rule of five were excluded, and the remaining compounds were subjected to further analyses.

### Virtual screening

Understanding the fundamental principles governing how protein receptors recognize, interact, and form associations with molecular substrates and inhibitors is crucial for drug discovery. PyRx v0.8 software ^16^ with an inbuilt AutoDock Vina 1.2.5 ^17^ for molecular docking was used to scan phytochemicals conforming to Lipinski’s rule of 5. AutoDock Vina uses a semi-empirical free-energy force field to predict the binding free energies of small molecules to macromolecular targets.

The human Androgen Receptor (PDB ID: 1E3G) was sourced from the RCSB Protein Data Bank. Initially, the protein structure underwent a curation process to remove any crystallographic water molecules and heteroatoms that might interfere with docking simulations. Subsequently, energy minimization was performed using UCSF Chimera vs 1.54 ( https://www.cgl.ucsf.edu/chimera/) to optimize the geometry of the protein. The steepest descent algorithm was applied for 100 steps, which is a common approach to relieve steric clashes and achieve a more stable conformation. Partial charges were then assigned to the protein using the AMBER ff14SB force field, which is well known for accurately modeling protein dynamics and interactions. The co-crystallized ligand metribolone (R18) was used as the control, and the ligands were docked at its active site.

### ADMET - ProTox II

The development of high-quality in silico ADMET models will enable compound efficacy and druggability features to be optimized concurrently, thereby improving the overall quality of drug candidates. ^18^ ProTox-II was used to experimentally validate the chemical toxicity and their combination. It uses machine learning models, the most common features, pharmacophore-based, fragment propensities, and chemical similarity to forecast different toxicity endpoints. ^19^ Based on the virtual screening results, the top ten phytochemical compounds were chosen for ADMET analysis.

### Induced fit docking

Induced fit docking was carried out using Schrodinger vs. 2020.3, which takes into account the flexibility of both the protein receptor and ligand, allowing for conformational changes to occur upon binding. The energy-minimized ligands were saved in PDB format for compatibility with the Schrodinger software, and the partial charges of the ligands were assigned, such as Gasteiger charges, which estimate the distribution of charges on the molecule based on its structure. Similarly, the protein charges may also be assigned using OPLS_2005 force fields to accurately capture its electrostatic properties. The grid box is a crucial parameter in docking simulations, as it defines the search space where the ligand can orient itself around the protein receptor. The dimensions of the grid box are typically specified in terms of the number of grid points along each axis (nx, ny, nz) and the grid spacing (Å) around the binding cavity residues LEU701, LEU707, MET742, MET745, ARG752, MET780, MET787, ALA748, LEU880, LEU873, PHE876, MET895, ILE899, THR877, GLN774, PHE764, LEU746, GLY708, GLN711, TRP741, ASN705. The dimensions were set to (58, 64, and 52 Å), providing a sufficient volume to explore potential binding modes of the ligand within the protein’s active site with a charge cutoff polarity set for a charge cutoff of 0.25 Å.

### Molecular dynamics simulation

Molecular dynamics (MD) simulations were conducted for the docked complex of the human Androgen Receptor with the best-docked molecule, employing Schrodinger Desmond 2020.1. ^20^ The OPLS-2005 force field, ^21^ along with an explicit solvent model using SPC water molecules, ^22^ were employed in this system. The simulation was performed in a periodic boundary solvation box with dimensions of 10 × 10 × 10 Å. To neutralize the charge, Na+ ions were added, and a 0.15 M NaCl solution was added to mimic the physiological environment. The initial equilibration was carried out using an NVT ensemble for 10 ns to allow the system to relax over the protein-ligand complexes. Subsequently, a short run of equilibration and minimization was conducted using an NPT ensemble for 12 ns. The NPT ensemble utilized the Nose-Hoover chain coupling scheme ^23^ with a temperature set at 37 °C, relaxation time of 1.0 ps, and pressure maintained at 1 bar in all simulations. A time step of 2 fs was used.

Pressure control was achieved using the Martyna-Tuckerman-Klein chain coupling scheme ^24^ with a relaxation time of 2 ps. The long-range electrostatic interactions were calculated using the particle mesh Ewald method, ^25^ and the Coulomb interaction radius was fixed at 9 Å. A RESPA integrator with a time step of 2 fs was used for each trajectory to calculate the bonded forces. The final production run was extended for 100 ns for the Human Androgen Receptor with the best-docked molecule complex. To track the stability of the MD simulations, a variety of parameters were computed, including the number of hydrogen bonds, radius of gyration (Rg), root-mean-square fluctuation (RMSF), and root-mean-square deviation (RMSD).

### Binding free energy analysis

Molecular Mechanics Generalized Born Surface Area (MM-GBSA) approaches are less computationally intensive than biochemical free energy methods and more precise than most molecular docking scoring systems. This method is useful for predicting the binding free energy in molecular systems. MM-GBSA is a useful technique for comprehending the impact of mutations on large biomolecular systems. ^26^ Biomolecular research has been utilized in investigations of protein folding, protein-ligand binding, protein-protein interactions etc. ^27^

The MM-GBSA approach was used to determine the binding free energies of the ligand-protein complexes. The MM-GBSA binding free energy was computed using the Python script thermal mmgbsa.py in the simulation trajectory with the VSGB solvation model and OPLS5 force field over the last 50 frames with a 1 step sampling size. The binding free energy of MM-GBSA (kcal/mol) was calculated using the additivity principle, wherein the differences in free energies, GBSA solvation energies, and surface area energies of ligand-protein complexes compared to their respective total energies of them individually were calculated.

## Results

### Differentially expressed genes (DEGs) analysis

Gene expression in TNBC and normal microarray datasets was compared to assess the underlying molecular pathways driving TNBC, and further network analysis was performed. Boolean operators and relevant filters were used to filter the microarray datasets using the GEO2R. The Benjamini-Hochberg-Yekutieli approach was used to adjust the P-value for the DEGs, and only the top 10% of the upregulated genes (P-value < 0.05) were selected. Tables 1 and 2 display the list of elevated genes with LogFC > 1.25 and P-value < 0.05 in dataset GSE45498 and GSE214101, respectively.

**Table 1.** The list of upregulated genes in dataset GSE45498 with LogFC > 1.25 and P-value <0.05.

Gene ID	Description	log _2_FC	p-Value
ESR1	Estrogen Receptor 1	3.45098	8.51E-14
IGFBP6	Insulin-like growth factors binding protein-6	3.115311	1.71E-14
NGFR	Nerve growth factor receptor	3.069617	3.26E-10
DLC1	Deleted in liver cancer 1	2.833933	1.03E-12
TGFBR3	Transforming Growth Factor Beta Receptor 3	2.631049	2.84E-10
EGR1	Early growth response factor 1	2.31673	5.84E-11
NTRK2	Neurotrophic Tyrosine Receptor Kinase	2.19261	1.77E-06
PPARG	Peroxisome proliferator-activated receptor gamma	2.151492	3.32E-10
CD34	CD34	1.887035	5.93E-09
IGF1	Insulin-Like Growth Factor-1	1.870246	1.53E-10
FOS	FOS	1.734574	5.27E-08
CAV1	Caveolin 1	1.694425	6.72E-07
FGF2	Fibroblast Growth Factor 2	1.61343	4.41E-04
KIT	KIT	1.547563	2.93E-05
AR	Androgen Receptor	1.381295	2.51E-04

**Table 2.** The list of upregulated genes in dataset GSE214101 with LogFC > 1.25 and P-value <0.05.

Gene ID	Description	log _2_FC	p-value
CDH4	cadherin 4	2.805	2.26E-06
MAP 2K6	mitogen-activated protein kinase kinase 6	2.659	2.16E-16
SHANK2	SH3 and multiple ankyrin repeat domains 2	2.62	7.80E-08
NEGR1	neuronal growth regulator 1	2.388	2.80E-03
AKAP6	A-kinase anchoring protein 6	2.26	7.91E-04
AR	androgen receptor	2.15	7.05E-02
MAP 2	microtubule associated protein 2	2.116	3.90E-08
NCAM2	neural cell adhesion molecule 2	2.091	2.00E-03
NLGN1	neuroligin 1	2.074	1.92E-04
ADGRL3	adhesion G protein-coupled receptor L3	2.049	1.37E-03
PRKG1	protein kinase cGMP-dependent 1	1.976	7.03E-05
PDE11A	phosphodiesterase 11A	1.895	1.30E-04
FAM78B	family with sequence similarity 78 member B	1.705	1.30E-04
PLXDC2	plexin domain containing 2	1.685	3.61E-11
SEMA3D	semaphorin 3D	1.657	2.45E-06
ID1	inhibitor of DNA binding 1	1.637	3.42E-03

**Figure 2.**
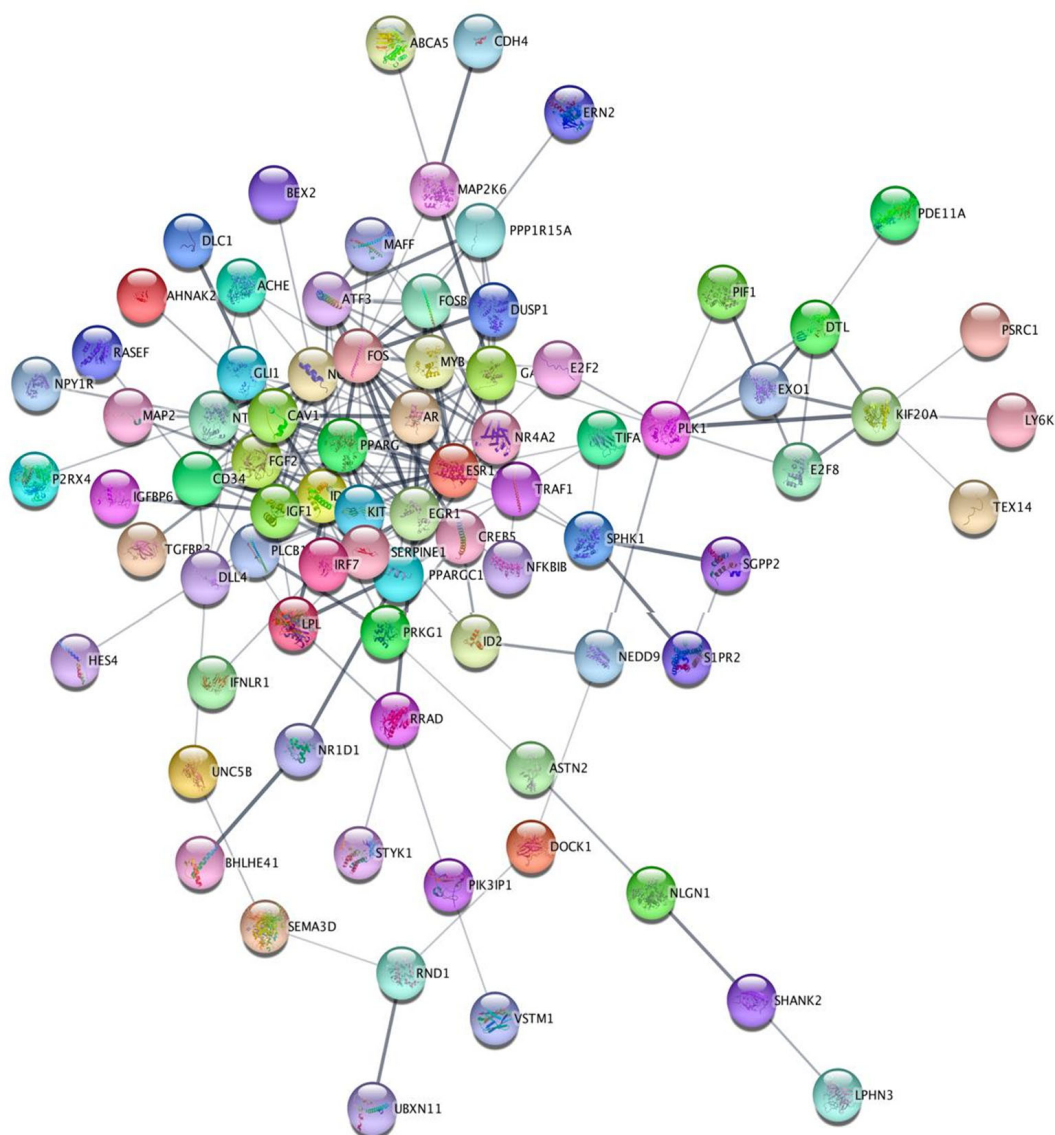
Protein–protein interaction network where Androgen receptor (AR) is the central hub gene.

The STRING tool was used to identify potential connections between DEGs in different tissues. ^12^ To build PPI networks, active interaction sources such as databases, co-expression, text mining, experiments, and species restricted to “Homo sapiens” were used, along with an interaction score greater than 0.4. The PPI network was displayed using Cytoscape v3.6.1 software as depicted in Figure 2.

**Table 3.** List of compounds used for Tox prediction.

Compound name	Docking score	Predicted LD _50_	Hepatotoxicity	Carcinogenicity	Immunotoxicity	Mutagenicity	Cytotoxicity
Chrysin 7-O-beta-D-glucopyranuronoside	-7.2	5000 mg/kg	Inactive	Inactive	Inactive	Inactive	Inactive
			0.73	0.51	0.96	0.74	0.81
Atalantoflavone	-7.1	2570 mg/kg	Inactive	Inactive	Inactive	Inactive	Inactive
			0.77	0.5	0.51	0.62	0.83
8-Prenyldaidzein	-6.8	2500 mg/kg	Inactive	Inactive	Inactive	Inactive	-
			0.7	0.66	0.8	0.65	
6-Prenylnaringenin	-6.7	2000 mg/kg	Inactive	Inactive	Inactive	Inactive	Inactive
			0.69	0.69	0.5	0.64	0.79
alpha-Isowighteone	-6.6	2875 mg/kg	Inactive	Inactive	Inactive	Inactive	Inactive
			0.71	0.61	0.85	0.55	0.81
2-Hydroxynaringenin	-6.5	2000 mg/kg	Inactive	Inactive	Inactive	Inactive	Inactive
			0.71	0.57	0.8	0.77	0.55
Carpachromene	-6.5	4000 mg/kg	Inactive	Inactive	Inactive	Inactive	Inactive
			0.77	0.5	0.61	0.62	0.83
8-Demethyleucalyptin	-6.3	3919 mg/kg	Inactive	Inactive	Inactive	Inactive	Inactive
			0.71	0.54	0.83	0.73	0.93
5-Hydroxy-7-acetoxy-8-methoxyflavone	-6.3	5000 mg/kg	Inactive	Inactive	Inactive	Inactive	Inactive
			0.76	0.54	0.87	0.7	0.83
Apigenin	-6.3	2500 mg/kg	Inactive	Inactive	Inactive	Inactive	Inactive
			0.68	0.62	0.99	0.57	0.87

The MCode plugin was employed to identify the highly linked regions inside the PPI network, while the CentiScape plugin was utilized to calculate the network topology parameters. Using degree and betweenness as the primary parameters, hub genes were identified. A complete set of algorithms, called CentiScape, was used to analyze the centrality of the network nodes. It can calculate multiple centralities for weighted, directed, and undirected networks. ^28^ The human Androgen Receptor was determined to be an appropriate hub gene in the protein-protein interaction network consisting of DEG genes.

### Virtual screening of phytochemical library

The human Androgen Receptor (hAR), covering the C-terminal amino acid residues (1E3G) with the co-crystallized ligand metribolone (R18), consists of 263 amino acid residues arranged in a three-layered α-helical sandwich structure. The ligand-binding pocket is located within the hydrophobic cavity formed by helices. A total of 1358 compounds were initially identified through systematic literature search, and their structures were retrieved from the PubChem database. Of these, only 543 compounds met the criteria outlined by Lipinski’s rule of five. These 543 compounds were then selected for the initial virtual screening against human Androgen Receptor using PyRx, and their binding affinities were tabulated ^29^ (refer to extended data Table S1). The top 50 ranked compounds were subjected to ADMET analysis on the ProTox II server. Only the top 10 ranked compounds that showed favorable binding affinity towards hAR based on their docking interaction and ideal ADMET properties were chosen for further analysis. The initial docking results and ADMET properties are shown in extended data.

### Induced fit docking and the molecular interactions

Molecular interaction studies of the binding cavity of the human Androgen Receptor and molecules are listed in extended data This was compared with the co-crystallized ligand associated with hAR protein R18 and analyzed by Schrodinger-induced fit docking. The ligand 2-hydroxynaringenin demonstrated high affinity for flexible residues within the binding pocket of the Human Androgen receptor protein. The calculated free energy of binding (ΔG) was determined to be -8.59 kcal/mol, indicating a strong binding interaction. While couple of other molecules 8-Prenyldaidzein and 5-Hydroxy-7-acetoxy-8-methoxyflavone also exhibited significant binding with HAR having ΔG = -8.54 kcal/mol and -8.26 kcal/mol, respectively. The highest affinity with a low negative binding energy was observed for 2-hydroxynaringenin, where the ligand formed conventional hydrogen bonds with Leu704, Asn705, Gln711, Met745, Arg752, and Thr877. Leu707, Met780, Leu873, and Phe876 were found to be involved in pi-alkyl and alkyl interactions with the 2-Hydroxynaringenin ligand. The binding energies of 2-Hydroxynaringenin and protein-ligand interactions are displayed in Figure 4 and the binding energies of other molecules are depicted in extended data.

**Figure 3.**
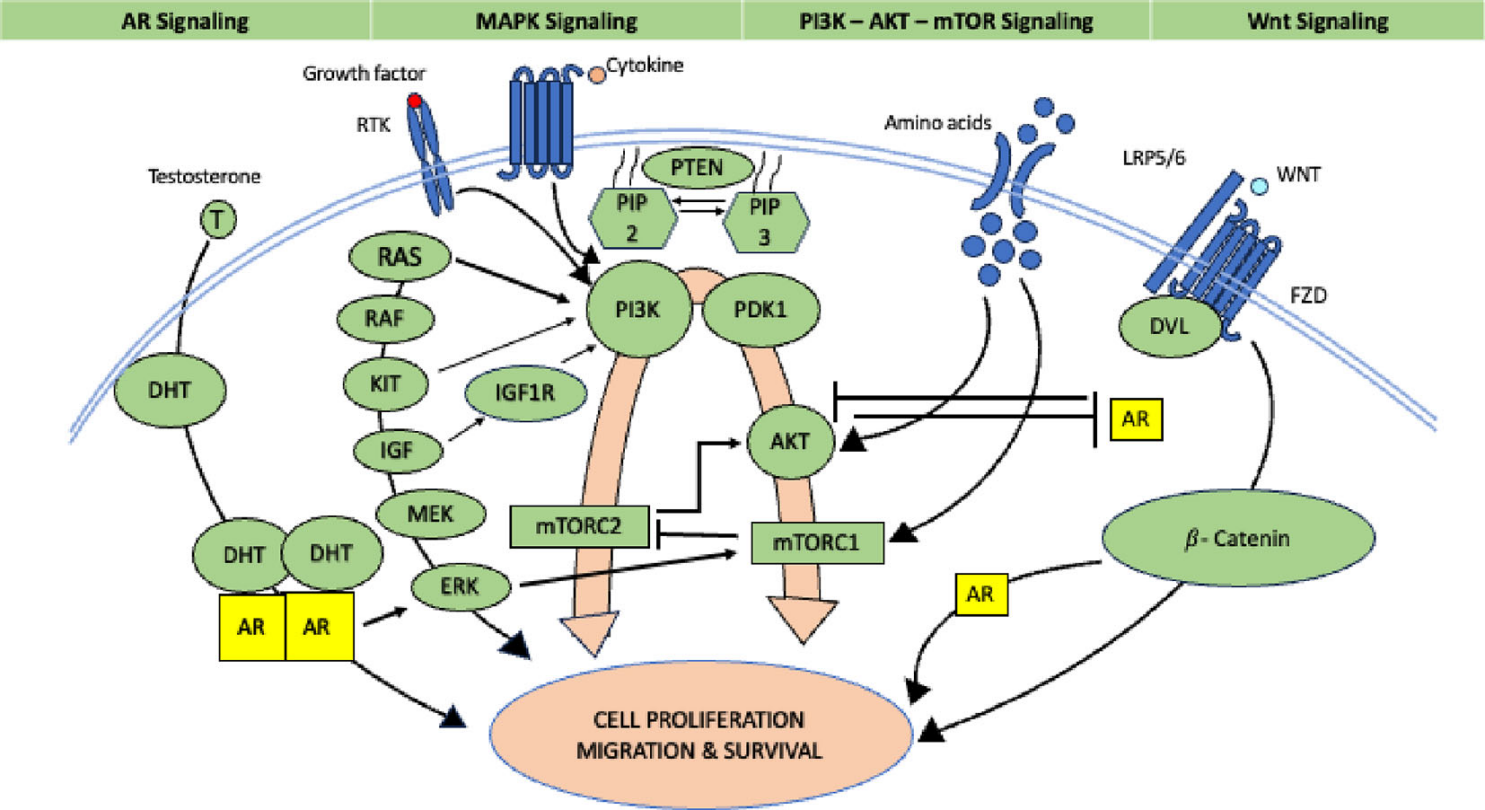
Role of Androgen receptor (Source modified from Ref. 30).

**Figure 4.**
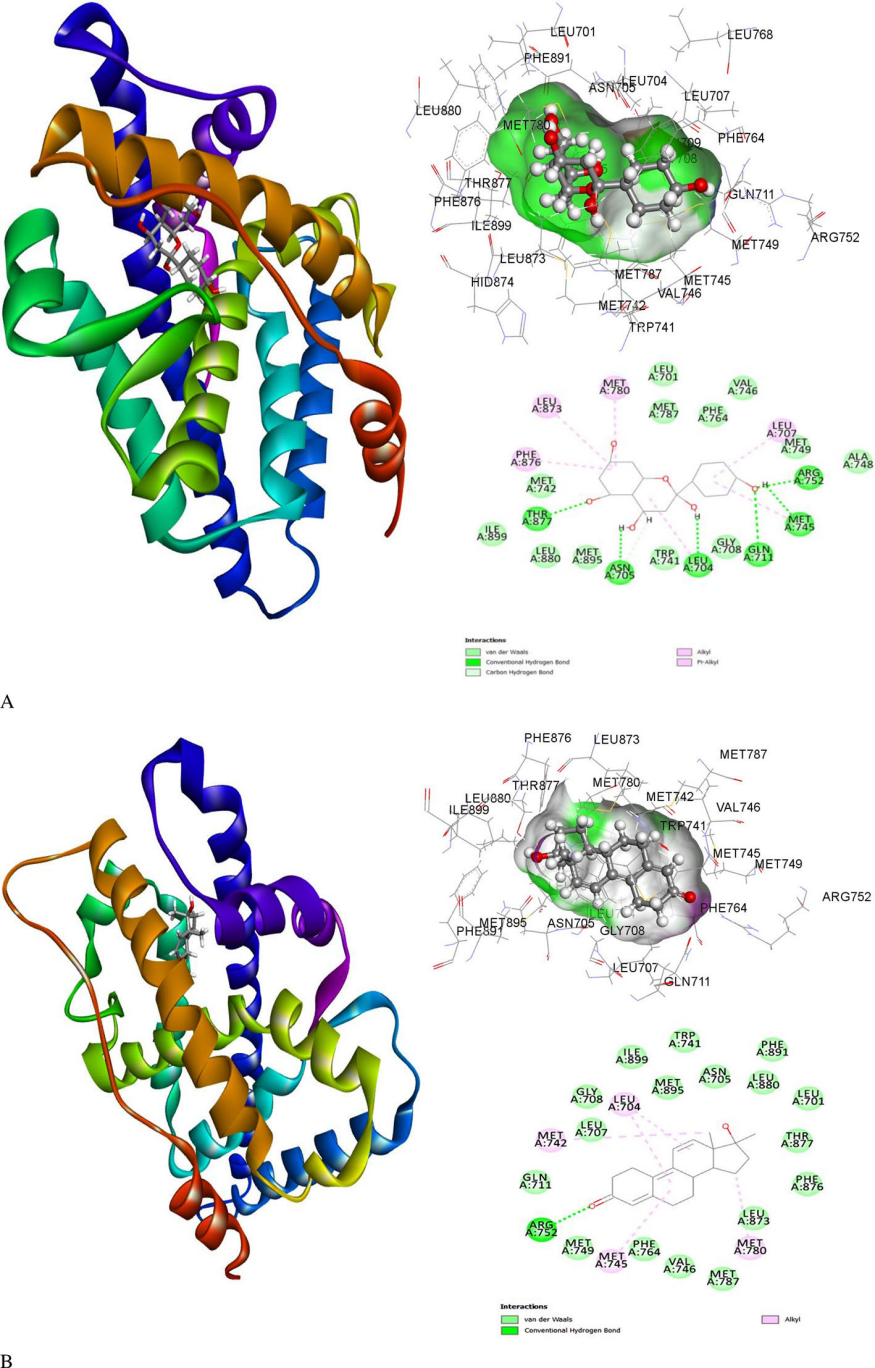
Induced fit docking pose of the ligand (A) 2-Hydroxynaringenin and co-crystallized (B) R18 molecules with HAR (PDB ID: 1E3G) displaying the ribbon shaped 3D protein and ligand interaction, 3D image of binding cavity residues and 2D interaction profile of bidning cavity residues with the respective ligands.

### Molecular dynamics simulation studies

**Figure 5.**
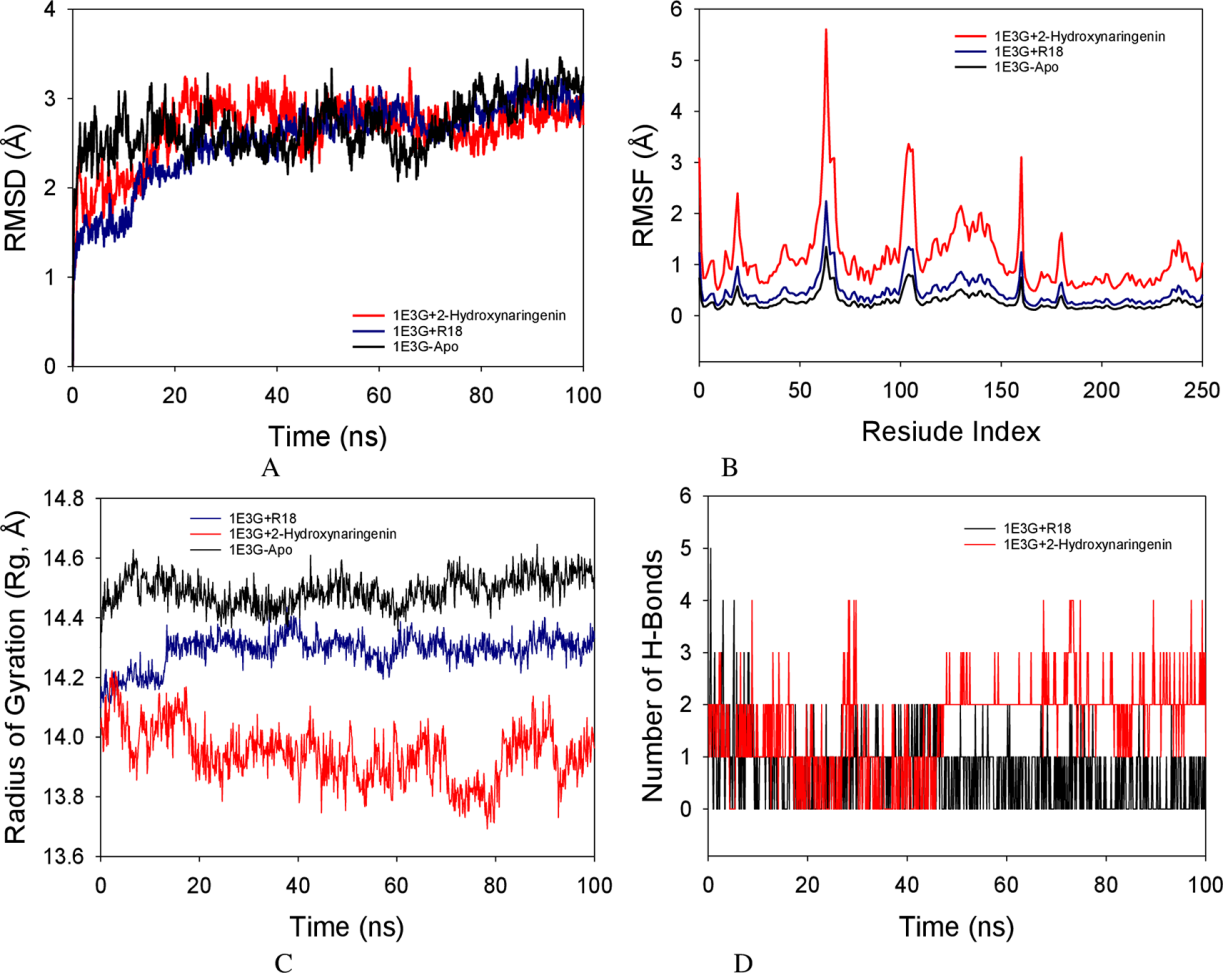
MD simulation analysis of 100 ns trajectories of (A) Cα backbone RMSD of 1E3G+2-Hydroxynaringenin (red), RMSD of 1E3GApo (black), and 1E3G+R18 (blue) (B) RMSF of Cα backbone RMSD of 1E3G+2-Hydroxynaringenin (red), RMSD of 1E3GApo (black), and 1E3G+R18 (blue) (C) Radius of gyration (Rg) of Cα backbone of Cα backbone RMSD of 1E3G+2-Hydroxynaringenin (red), RMSD of 1E3GApo (black), and 1E3G+R18 (blue) (D) Formation of hydrogen bonds in 1E3G+2-hydroxynaringenin (red) and R18 (black).

Molecular dynamics simulation (MD) investigations were performed to ascertain the convergence and stability of 1E3G-Apo (no ligand hAR protein), 1E3G+R18 (R18 co-crystallized ligand) and 1E3G+2-Hydroxynaringenin complexes. When comparing the root mean square deviation (RMSD) measurements, the 100 ns simulation showed a stable conformation. The Apo protein’s Cα-backbone’s RMSD showed a 3.0 Å divergence ( Figure 5A). While 1E3G+R18 and 1E3G+2-Hydroxynaringenin both showed 2.9 Å, the overall RMSD is shown to be 2.9 Å ( Figure 5A).

The root mean square fluctuations (RMSF) plot of the 1E3G+2-Hydroxynaringenin complex protein revealed notable variations at residues 60–70, 110–120, and 180–185, which may have been caused by the residues’ increased flexibility. The rest of the residues fluctuated less during the course of the 100 ns simulation ( Figure 5B). Radius of gyration (Rg) in this study, 1E3G Cα-backbone bound to Apo protein displayed increment of Rg values indicating lesser compactness while stable Rg was observed from 20.2 to 20.3 Å in 1E3G+R18 ( Figure 5C). The number of hydrogen bonds was significantly different between 1E3G+2-Hydroxynaringenin, throughout the simulation time of 100 ns ( Figure 5D). The average number of hydrogen bonds observed in 1E3G+2-Hydroxynaringenin was two on average in MD simulation studies ( Figure 5D, red color).

### Mechanics generalized born surface area (MM-GBSA) calculations

The binding free energy and other contributing energies in the form of MM-GBSA were found for HAR+2-hydroxynaringenin by using the MD simulation trajectory. According to results ( Table 4), the simulated complexes’ stability was primarily attributed to ΔGbindCoulomb, ΔGbindvdW, and ΔGbindLipo, whereas ΔGbindCovalent and ΔGbindSolvGB contributed to the corresponding complexes’ instability.

**Table 4.** Binding free energy components for the 1E3G+2-hydroxynaringenin and 1E3G+R18 calculated by MM-GBSA.

Energies (kcal/mol)	1E3G+2-hydroxynaringenin	1E3G+R18
**ΔG**_**bind**_	-31.53±5.3	-29.95±4.1
**ΔG**_**bind**_ **Lipo**	-29.83±3.2	-23.51±3.2
**ΔG**_**bind**_ **vdW**	-22.68±3.22	-16.27±1.21
**ΔG**_**bind**_ **Coulomb**	-5.22±2.11	-7.45±2.8
**ΔG**_**bind**_ **H**_**bond**_	-0.9±0.1	-0.6±0.2
**ΔG**_**bind**_ **SolvGB**	33.91±1.27	41.27±1.76
**ΔG**_**bind**_ **Covalent**	0.79±0.3	1.24±0.23

## Discussion

The integrated analysis of gene expression and protein-protein interactions (PPI) would help to identify candidates that could serve as therapeutic targets. In this study, we compared TNBC datasets to normal datasets to assess the underlying molecular pathways that drive TNBC. Differential gene expression profiling of the selected datasets using the Benjamini-Hochberg-Yekutieli approach was used to adjust the P-value, which controls the rate of false discovery under positive dependence assumptions. Then, using STRING, which incorporates both known and anticipated PPIs, the protein-protein interactions between the previously mentioned genes were investigated using Cytoscape. CentiScape was used to analyze the centrality of network nodes, and the Human Androgen Receptor was determined to be an appropriate hub gene in the protein-protein interaction network consisting of DEG genes.

The Androgen Receptor (AR) pathway is becoming a viable therapeutic target in breast cancer. 12-55% of TNBC cases, which provides a chance for targeted treatment. The “Luminal AR (LAR)” molecular subtype of TNBC is where AR is most prevalent. The LAR subtype exhibits the highest amount of AR expression amongst the many molecular subtypes of TNBC in which it is present. All AR+ TNBC primary tumors that were evaluated showed nuclear localization of AR, a sign of transcriptionally active receptors. Many investigations have shown that AR expression in breast cancer, particularly in the TNBC subtype, has been linked to an overall better outcome. Considering that > 70% of AR expression is consistent between primary and metastatic breast cancers, AR may be a novel diagnostic and therapeutic target for patients with AR-positive breast cancer. In luminal mammary carcinomas, a high percentage of cases express androgen receptors (AR), and the ratio of AR to estrogen receptors (ER) or progesterone receptors (PR) is considered a potential prognostic factor. However, in estrogen receptor-negative (ER-) tumors, AR expression is associated with a poorer prognosis. Androgen receptor (AR) expression has demonstrated predictive value for potential response to adjuvant hormonal therapy in estrogen receptor-positive (ER+) breast cancers. Additionally, AR expression has been associated with predicting responses to neoadjuvant chemotherapy in triple-negative breast cancer (TNBC). The role of the AR is shown in Figure 3.

The human Androgen Receptor (hAR), which has 920 amino acid residues, was identified as the primary therapeutic target for triple-negative breast cancer. The 3D crystal of human AR retrieved from the Protein Data Bank (PDB ID 1E3G) is a partial structure covering the C-terminal amino acid residues 658-920. This region encompasses the nuclear receptor ligand-binding domain (NR LBD) of hAR. It consists of 263 amino acid residues, arranged in a three-layered α-helical sandwich structure. The ligand-binding pocket is located within the hydrophobic cavity formed by helices. Virtual screening of 543 ligands against human AR was performed using PyRx at the co-crystallized ligand-binding site. The top 10 ranked compounds that showed favorable binding affinity towards hAR and ideal ADMET properties were chosen for induced fit docking.

Unlike rigid docking, induced fit docking treats the ligand and protein as typically flexible entities allowing for conformational changes to occur upon binding. The ligand 2-hydroxynaringenin demonstrated a high affinity for the flexible residues within the binding pocket of hAR, with an interaction binding energy of-8.59 kcal/mol with six conventional hydrogen bonds, indicating a strong binding interaction. Interestingly, the interaction binding energy of the hAR protein with R18 was observed to be -7.8 kcal/mol and only one conventional hydrogen bond formed between R18 and Arg752 ( Figure 4). No other potential interactions were observed, except for van der Waal’s instructions. For both 2-Hydroxynaringenin and R18, it was observed that Arg752 is the key residue for ligand binding and could play an active role in protein function.

Molecular dynamics (MD) simulation studies of 100 ns showed stable conformations with 1E3G+2-Hydroxynaringenin complexes. The RMSD of the Cα-backbone of the Apo protein exhibited a deviation of 3.0 Å. While 1E3G+R18 exhibited 2.9 Å and simlarly 1E3G+2-Hydroxynaringenin also exhibited the total RMSD is depicted to be 2.9 Å ( Figure 5A). All RMSD values were below the acceptable range of 3 Å. Stable RMSD plots of apo-1E3G, 1E3G+R18 and 1E3G+2-Hydroxynaringenin were observed to be less than 3 Å. Therefore, it can be suggested that apo-1E3G, 1E3G+R18 and 1E3G+2-Hydroxynaringenin complexes are well converged and equilibrated.

The RMSF of the 1E3G+2-Hydroxynaringenin complex protein exhibited notable fluctuation spikes at residues 60–70, 110–120, and 180–185, which may have been brought on by the residues’ increased flexibility. During the course of the 100 ns simulation, the remaining residues fluctuated less. A more rigid conformation with fewer fluctuations was observed in the Apo-protein and 1E3G+R18 complex. Therefore, from the RMSF plots, it can be suggested that the structures of 1E3G+2-Hydroxynaringenin are more flexible during simulation in ligand-bound conformations. The radius of gyration (Rg) is a measure of protein compactness. Lowering and stable of radius of gyration (Rg) from 20.0 to 20.02 Å in 1E3G+2-Hydroxynaringenin was observed. The quantity of hydrogen bonds forming between the ligand and protein indicates a strong connection and stability of the complex. Over the course of the 100 ns simulation, there was a considerable difference in the amount of hydrogen bonds between 1E3G+2-Hydroxynaringenin ( Figure 5D). The average number of hydrogen bonds observed in 1E3G+2-Hydroxynaringenin was two on average in MD simulation studies ( Figure 5D, red).

Using the MD simulation trajectory, the binding free energy and additional contributing energies in the form of MM-GBSA were found for HAR+2-hydroxynaringenin. The findings ( Table 4) show that ΔGbindCoulomb, ΔGbindvdW, and ΔGbindLipo were the main contributors to ΔGbind in the simulated complexes’ stability, whereas ΔGbindCovalent and ΔGbindSolvGB were responsible for the corresponding complexes’ instability. HAR+2-hydroxynaringenin complex showed significantly higher binding free energies. The capacity of 2-hydroxynaringenin to bind to the chosen protein efficiently and form stable protein-ligand complexes was demonstrated by these data, which further validated the compound’s potential.

## Conclusion

In recent years, bioinformatic analysis has become essential for studying the pathogenesis of human diseases. Differential gene expression studies, protein–protein interactions, and network topology analyses were performed. The current study identified the human Androgen Receptor (AR) as a potential drug target to combat triple-negative breast cancer (TNBC). This was concluded based on gene expression profiling, protein-protein interaction, and network topology analysis. The specific role of the Androgen Receptor in breast cancer growth and progression remains uncertain, although the AR is expressed in approximately 77% of all breast cancers, even higher than Estrogen Receptors (ERs). ^31^ A more luminal, well-differentiated, and less aggressive tumor may be indicated by high expression of Androgen Receptor in breast cancer, which could improve prognosis. ^32^ AR inhibition tends to be well-tolerated, and patients with TNBC may benefit from it when paired with other medications, as its toxicity is much lower than that of chemotherapy. Combinations involving mTOR inhibitors, EGFR and other ErbB inhibitors, PIK3 inhibitors, anti-PDL1 antibodies, paclitaxel, and other chemotherapeutic drugs are supported by preclinical results. Randomized clinical trials would be required to ascertain the clinical utility of AR inhibitors. ^33^^–^^35^

Flavonoids are a class of natural compounds found in various fruits, vegetables, and plants and have been extensively studied for their potential therapeutic effects, including their ability to combat cancer. Naringenin, specifically categorized as a flavanone, is a flavonoid present in grapefruit and tomatoes, among other dietary sources. ^36^ The antioxidant and anti-inflammatory properties of naringenin have led to its exploration for various potential use in the pharmaceutical industry. ^37^

### Limitations of the study

The current study identified the human Androgen Receptor as a potential candidate drug target to combat TNBC and recognized 2-hydroxynaringenin as a potential lead molecule. The in vitro and in vivo efficacies of 2-hydroxynaringenin require further investigation. Safety, pharmacokinetics, and pharmacodynamics tests need to be performed to further develop hydroxynaringenin for clinical use.

## Data Availability

1.
GEO DATASET 1 - Accession number- GSE45498 *https://www.ncbi.nlm.nih.gov/geo/query/acc.cgi?acc=GSE45498*

Platform–GPL16299 2.
GEO DATASET 2 - Accession number- GSE214101 *https://www.ncbi.nlm.nih.gov/geo/query/acc.cgi?acc=GSE214101* Supplementary data 1. Figshare: Molecular docking and MD simulation approach to identify potential phytochemical lead molecule against triple negative breast cancer - Supplementary Figures.docx - DOI: 10.6084/m9.figshare.26967880.v1 ^38^
Data are available under the terms of the Creative Commons Zero “No rights reserved” data waiver (CC0 1.0 Public domain dedication). 2. Figshare: Molecular docking and MD simulation approach to identify potential phytochemical lead molecule against triple negative breast cancer - Supplementary Table.docx - DOI: 10.6084/m9.figshare.26967733.v1 ^39^
Data are available under the terms of the Creative Commons Zero “No rights reserved” data waiver (CC0 1.0 Public domain dedication).
