# Supplementary material for: Molecular docking and MD simulation approach to identify potential phytochemical lead molecule against triple negative breast cancer
Source: F1000Res. 2025 Mar 18;13:1271. Originally published 2024 Oct 24. [Version 2] doi: 10.12688/f1000research.155657.2 (PMC12375911; doi:10.12688/f1000research.155657.2)
Supplement: Peer review history

# Reviewer report: Reviewer response for version 2

**DOI:** 10.5256/f1000research.178643.r371506

**Published:** 2025-08-24

**Authors:** Shiek S S J Ahmed

**Affiliations:** 1 Chettinad Academy of Research and Education, Kelambakkam 603103, Tamil Nadu,, India

The author responded to all comments. If there is no negative report from other reviewers, it can be accepted

Is the work clearly and accurately presented and does it cite the current literature?

Yes

If applicable, is the statistical analysis and its interpretation appropriate?

I cannot comment. A qualified statistician is required.

Are all the source data underlying the results available to ensure full reproducibility?

Yes

Is the study design appropriate and is the work technically sound?

Partly

Are the conclusions drawn adequately supported by the results?

Partly

Are sufficient details of methods and analysis provided to allow replication by others?

Yes

Reviewer Expertise:

Neuroscience, Cancer research, immunoinformatic, systems biology, NGS, artificial intelligence, omics research, Big data analytics

I confirm that I have read this submission and believe that I have an appropriate level of expertise to confirm that it is of an acceptable scientific standard.

# Reviewer report: Reviewer response for version 2

**DOI:** 10.5256/f1000research.178643.r393020

**Published:** 2025-07-25

**Authors:** Indiraleka Muthiah

**Affiliations:** 1 Mepco Schlenk Engineering College, Sivakasi, Tamil Nadu, India

The research titled “Molecular docking and MD simulation approach to identify potential phytochemical lead molecule against triple negative breast cancer” effectively presents an in silico strategy to identify potential phytochemical ligands targeting TNBC molecular markers. The study concludes that the androgen receptor (AR) is a promising target and highlights 2-Hydroxynaringenin as a potential lead molecule for AR. The manuscript is well-written, with a clearly defined methodology that effectively addresses the research objective. However, the following suggestions could enhance the manuscript further:

1. The significance of the current research should be emphasised in the background section of the abstract to convey its impact and relevance.
2. Numerous molecular targets and phytochemical ligands for TNBC have been explored through in silico studies. In the introduction, it would strengthen the manuscript to differentiate this study from existing research by outlining its novelty and contribution, supported by up-to-date literature. Additionally, comparing the current results with previously reported studies would provide valuable context.
3. The ligand selection process requires more detailed explanation, including the specific criteria and rationale for choosing the phytochemicals investigated. Since many phytochemicals have been studied against breast cancer, clarifying why only a limited subset was selected here will add clarity.
4. The docking results section could benefit from identifying and discussing the binding sites, particularly in comparison to standard AR inhibitors. The explanation of molecular docking outcomes is currently insufficient, making it challenging to interpret and compare the ligands presented in Table 3. Clarification on whether the ligands are ranked by binding affinity and the reasoning behind selecting 2-Hydroxynaringenin for further study should be included.
5. The discussion section should be expanded to provide a more comprehensive interpretation of the findings, leading to clearer conclusions.

It is important to note that while this in silico approach can propose potential lead molecules for AR, confirmation of their efficacy and potential requires further validation through in vitro and in vivo assays.

Is the work clearly and accurately presented and does it cite the current literature?

Partly

If applicable, is the statistical analysis and its interpretation appropriate?

I cannot comment. A qualified statistician is required.

Are all the source data underlying the results available to ensure full reproducibility?

Yes

Is the study design appropriate and is the work technically sound?

Yes

Are the conclusions drawn adequately supported by the results?

Partly

Are sufficient details of methods and analysis provided to allow replication by others?

Yes

Reviewer Expertise:

Research on the design and delivery of drugs for TNBC

I confirm that I have read this submission and believe that I have an appropriate level of expertise to confirm that it is of an acceptable scientific standard, however I have significant reservations, as outlined above.

# Reviewer report: Reviewer response for version 2

**DOI:** 10.5256/f1000research.178643.r372224

**Published:** 2025-04-25

**Authors:** Yutian Zou

**Affiliations:** 1 Sun Yat-sen University Cancer Center, Guangzhou, China

The manuscript titled *“Molecular docking and MD simulation approach to identify potential phytochemical lead molecule against triple negative breast cancer”* presents an in silico approach to identify novel therapeutic candidates for triple-negative breast cancer (TNBC). Using differential gene expression analysis and protein-protein interaction networks, the androgen receptor (AR) was identified as a potential target. Subsequent virtual screening and molecular dynamics simulations suggested that 2-hydroxynaringenin may serve as a promising phytochemical inhibitor of AR. The study demonstrates the structural stability and favorable binding affinity of the 2-hydroxynaringenin–AR complex through MM-GBSA calculations. However, the following issues are required for explaining:

1. The computational identification of 2-hydroxynaringenin as a potential AR inhibitor is interesting, but it is essential to experimentally validate the binding affinity and functional relevance of this interaction in vitro. Cell-based assays using AR-expressing TNBC cell lines should be conducted to confirm the direct binding and biological effect of 2-hydroxynaringenin.
2. The inhibitory effect of 2-hydroxynaringenin on AR activity should be quantified using standard biochemical assays to determine its IC₅₀ value. This quantitative measurement is crucial to evaluate its potency compared to known AR inhibitors.
3. The authors should consider validating their docking and simulation workflow by applying it to known AR inhibitors such as enzalutamide, bicalutamide, and apalutamide. Comparing the binding affinity and stability of these compounds with 2-hydroxynaringenin using the same computational pipeline is important to demonstrate the predictive power of the proposed approach.
4. The manuscript would benefit from a more detailed discussion on how the computational findings could inform future drug development. Specifically, how can the identified phytochemical be optimized or modified for enhanced efficacy and bioavailability? What are the steps for translating this lead compound into preclinical or clinical testing?
5. Essential details of data and analysis in this study remain unclear. For example, there is a lack of details on how docking data were processed and normalized? A reproducible detail should be provided.
6. Some similar studies regarding the docking and cancer should be cited and discussed. For example, PMID: 39912365, 39081282.
7. The current study lacks a discussion on the limitations of in silico-only studies. The authors should acknowledge the absence of experimental validation and outline clear next steps, including in vitro assays, pharmacokinetic evaluation, and possible structural modification of the lead compound to improve drug-like properties.
8. The authors are recommended to consider engaging a professional language editing service to ensure the clarity and coherence of the manuscript.

Is the work clearly and accurately presented and does it cite the current literature?

Partly

If applicable, is the statistical analysis and its interpretation appropriate?

Yes

Are all the source data underlying the results available to ensure full reproducibility?

Partly

Is the study design appropriate and is the work technically sound?

Partly

Are the conclusions drawn adequately supported by the results?

Partly

Are sufficient details of methods and analysis provided to allow replication by others?

Partly

Reviewer Expertise:

breast cancer

I confirm that I have read this submission and believe that I have an appropriate level of expertise to confirm that it is of an acceptable scientific standard, however I have significant reservations, as outlined above.

## References

[1] : Design, Synthesis and Molecular Docking of New Thieno[2,3‑d]Pyrimidin-4-One Derivatives as Dual EGFR and FGFR Inhibitors. *Drug Dev Res* .2025;86(1) : 10.1002/ddr.70061 e70061 10.1002/ddr.70061 39912365

[2] : RN486, a Bruton's Tyrosine Kinase inhibitor, antagonizes multidrug resistance in ABCG2-overexpressing cancer cells. *J Transl Int Med* .2024;12(3) : 10.2478/jtim-2024-0011 288-298 10.2478/jtim-2024-0011 39081282 PMC11284896

# Reviewer report: Reviewer response for version 1

**DOI:** 10.5256/f1000research.170851.r335168

**Published:** 2024-12-10

**Authors:** Shiek S S J Ahmed

**Affiliations:** 1 Chettinad Academy of Research and Education, Kelambakkam 603103, Tamil Nadu,, India

This manuscript provides valuable insights into drug discovery in the field of oncology. The authors have done excellent work in exploring breast cancer treatment by utilizing efficient computational methods such as differential gene expression, protein network, molecular docking, molecular dynamics simulations, and MM-GBSA. The study aims to identify potential lead compounds against a candidate target for triple-negative breast cancer. However, the following issues need to be addressed before indexing.

 1. Abstract needs to reframed {

 ( **#Note**: Can be re-constructed based to authors preference)

 Example:

**Background**

 Triple-negative breast cancers (TNBC) are defined as tumors that lack the expression of the estrogen receptor (ER), progesterone receptor (PR), and human epidermal growth factor receptor 2 (HER2). It exhibits unique clinical and pathological features, high aggressiveness, and has a relatively poor prognosis and clinical outcome.

**Objective**

 To identify a novel drug target protein against TNBC and potential phytochemical lead molecules against the identified targets.

**Methods**

 In this study, we retrieved TNBC samples from NGS and microarray datasets in the Gene Expression Omnibus database. We employed a combination of differential gene expression studies, protein-protein interaction analysis, and network topology investigation to identify the target protein. Additionally, the molecular docking and molecular dynamics (MD) simulation studies followed by Molecular Mechanics with Generalised Born Surface Area salvation was used to identify potential lead molecule.

**Results**

 The upregulated genes with LogFC > 1.25 and P-value < 0.05 from the TNBC gene expression dataset were identified. Androgen receptor (AR) was found to be an appropriate hub target in the protein-protein interaction network. Phytochemicals that inhibit breast cancer target were retrieved from the PubChem database and virtual screening was performed using PyRx against the AR protein. Thereby, the AR was found to be the target protein and 2-hydroxynaringenin was discovered to be a possible phytochemical lead molecule for combating TNBC. Moreover, the AR and the 2-hydroxynaringenin complex showed structural stability and higher binding affinity through molecular dynamics and MM-GBSA studies.

**Conclusion**

 AR was identified as a hub protein that is highly expressed in breast cancer and 2-hydroxynaringenin efficacy of counter TNBC requires further investigation both in vitro and in vivo.} please refer to following link for more details https://f1000research.s3.amazonaws.com/linked/695061.155657-F1000_review_1_.docx

 2. Please do not use scientific term multiple times. rather use the abbreviation.

 3. In the methodology section, the calculation of MD trajectories is unclear. Was the Desmond plug-in employed, or manually? Additionally, there appears to be a contrast between the described MD simulation methodology and the reported results. The MD simulation results might have been obtained using GROMACS rather than Desmond. This assumption arises because Desmond typically calculates parameters such as the Rg and intramolecular hydrogen bonds (intraHB) for the ligand's extendness and internal hydrogen bonding, but not for the entire complex.

 4. The authors should clearly define the criteria used for selecting those two datasets (gene expression) in the methodology section. The datasets seems heterogeneous one consists of human sample (expression array) while the other consists of cell line samples (RNA-sequencing). How the authors address and account for batch effects arising from these differences?

 4a. The basis for the adopting the LogFC > 1.25 as a threshold while in gene expression analysis should be explained

 5. How the 2D interaction of protein and ligand was visualized after docking.

 6. In Fig. 5B, the RMSF does not cover the entire protein (263 amino acids).

 7. Some important references are missing in the discussion section, particularly in AR association with Breast cancer (second paragraph).

 8. In Fig 2, highlighting the hub target protein or constructing the sub-network by centering AR would enhance visualization and helpful for the new readers to understand. 

 9. The following sentence needs referencing “All RMSD values were below the acceptable range of 3Å”.

 10. The interpretation of the MM-GBSA results needs to be clarified. Specifically, the outcomes of the MM-GBSA analysis comparing the test and control compounds should be explained in detail.

Is the work clearly and accurately presented and does it cite the current literature?

Yes

If applicable, is the statistical analysis and its interpretation appropriate?

I cannot comment. A qualified statistician is required.

Are all the source data underlying the results available to ensure full reproducibility?

Yes

Is the study design appropriate and is the work technically sound?

Partly

Are the conclusions drawn adequately supported by the results?

Partly

Are sufficient details of methods and analysis provided to allow replication by others?

Yes

Reviewer Expertise:

Neuroscience, Cancer research, immunoinformatic, systems biology, NGS, artificial intelligence, omics research, Big data analytics

I confirm that I have read this submission and believe that I have an appropriate level of expertise to confirm that it is of an acceptable scientific standard, however I have significant reservations, as outlined above.

# Response

**Published:** 2025-02-06

**Authors:** Pranaya Sankaranarayanan

**Affiliations:** Bioinformatics, Sri Ramachandra Institute of Higher Education and Research (Deemed to be University), Chennai, Tamil Nadu, India

1. Abstract needs to reframed

**Response: ** Thank you for your suggestion. The abstract has been restructured.

**Background**

 Triple-negative breast cancers (TNBC) are defined as tumors that lack the expression of the estrogen receptor (ER), progesterone receptor (PR), and human epidermal growth factor receptor 2 (HER2). It exhibits unique clinical and pathological features, demonstrates high aggressiveness, and has a relatively poor prognosis and clinical outcome.

**Objective**

 To identify a novel drug target protein against TNBC and potential phytochemical lead molecules against the identified targets.

**Methods**

 In this study, we retrieved TNBC samples from NGS and microarray datasets in the Gene Expression Omnibus database. We employed a combination of differential gene expression studies, protein-protein interaction analysis, and network topology investigation to identify the target protein. Additionally, the molecular docking and molecular dynamics (MD) simulation studies followed by Molecular Mechanics with Generalised Born Surface Area salvation was used to identify potential lead molecule.

**Results**

 The upregulated genes with LogFC > 1.25 and P-value < 0.05 from the TNBC gene expression dataset were identified. Androgen receptor (AR) was found to be an appropriate hub target in the protein-protein interaction network. Phytochemicals that inhibit breast cancer target were retrieved from the PubChem database and virtual screening was performed using PyRx against the AR protein. Thereby, the AR was found to be the target protein and 2-hydroxynaringenin was discovered to be a possible phytochemical lead molecule for combating TNBC. Moreover, the AR and the 2-hydroxynaringenin complex showed structural stability and higher binding affinity through molecular dynamics and MM-GBSA studies.

**Conclusion**

 AR was identified as a hub protein that is highly expressed in breast cancer and 2-hydroxynaringenin efficacy of counter TNBC requires further investigation both in vitro and in vivo.

 2. Please do not use scientific term multiple times. rather use the abbreviation.

**Response:** Thank you for the input. The changes have been made.

 3. In the methodology section, the calculation of MD trajectories is unclear. Was the Desmond plug-in employed, or manually? Additionally, there appears to be a contrast between the described MD simulation methodology and the reported results. The MD simulation results might have been obtained using GROMACS rather than Desmond. This assumption arises because Desmond typically calculates parameters such as the Rg and intramolecular hydrogen bonds (intraHB) for the ligand's extendness and internal hydrogen bonding, but not for the entire complex.

**Response: ** In the methodology section it was quite clearly mentioned that Desmond 2020.1 engine was used manually for the study. Moreover, the concern was raised the superiority of GROMACS over Desmond is somehow confusing and as per the suggestion in order to obtain the exact parameters there are options such as simulation event analysis where the hydrogen bonding calculates between the protein and ligand complex. Desmond is also well documented and parameterized simulation engine like GROMACS and there are many reports and previous literatures successfully attributed the specificity of the tool. Fig. 5B, RMSF extracted from the tool outcome and regions having spikes with high and moderate significance exhibited in the figure. The outcome of MMGBSA is compared with the control R18 compound which was co-crytallized inhibitor in the pdb structure of the receptor.

 4. The authors should clearly define the criteria used for selecting those two datasets (gene expression) in the methodology section. The datasets seem heterogeneous one consists of human sample (expression array) while the other consists of cell line samples (RNA-sequencing). How the authors address and account for batch effects arising from these differences?

**Response: ** We appreciate your insightful comments regarding the dataset selection and batch effect considerations. In our study, we selected the two datasets—GSE45498 (microarray from human tissue samples) and GSE214101 (RNA-seq from cell lines)—based on their relevance to triple-negative breast cancer (TNBC) and their comprehensive gene expression profiles. These datasets were chosen to enable a broader investigation of TNBC-related gene expression variations across different biological contexts. To address potential batch effects arising from differences in platform technology and sample types, we implemented standard normalization and transformation techniques.

 4a. The basis for the adopting the LogFC > 1.25 as a threshold while in gene expression analysis should be explained

**Response: ** A log₂FC of 1.25 corresponds to a 2.4-fold change in expression, meaning the gene expression is increased by ~140% (if positive) or decreased to ~42% of its original level (if negative). Many biological processes, such as regulatory networks and signalling pathways, involve moderate gene expression changes rather than extreme shifts. Traditional thresholds like log₂FC ≥ 2 (4-fold change) are quite conservative and may exclude relevant genes, especially for genes with small but functional changes. A log₂FC threshold of 1.25 provides a balance—capturing biologically significant changes while avoiding detection of genes with minor fluctuations.

**Maintaining a balance between sensitivity and specificity**:

- Setting too high a threshold (e.g., log₂FC ≥ 2) might miss key regulatory genes with moderate but meaningful expression changes.
- A threshold of log₂FC ≥ 1.25, combined with an adjusted p-value (e.g., FDR ≤ 0.05), ensures that identified genes are statistically reliable and biologically relevant.

**Reducing false positives while capturing meaningful expression shifts**:

- Log₂FC values <1 (e.g., 0.5, which is ~1.4-fold change) might capture noise or small fluctuations due to experimental variability.
- Using 1.25 as a threshold ensures changes are more likely due to biological regulation rather than technical variation.

5. How the 2D interaction of protein and ligand was visualized after docking

**Response:** By using discovery studio, the 2D interaction of protein and ligand was visualized.

 6. In Fig. 5B, the RMSF does not cover the entire protein (263 amino acids).

**Response:** The 3D structure of Androgen Receptor (PDB ID: 1E3G) does not cover the entire protein and contains only the amino acids 671-920 (comprising of 250 amino acids). The manuscript also has been modified to reflect this.

 7. Some important references are missing in the discussion section, particularly in AR association with Breast cancer (second paragraph).

**Response: ** References have been added

 8. In Fig 2, highlighting the hub target protein or constructing the sub-network by centering AR would enhance visualization and helpful for the new readers to understand. 

**Response:** Thank you for your valuable suggestion. We have incorporated the changes.

https://f1000research.s3.amazonaws.com/linked/707287.155657-Comment_to_reviewer_Sheik_by_Author_Pranaya.pdf

 9. The following sentence needs referencing “All RMSD values were below the acceptable range of 3Å”.

**Response:** Reference has been added

 10. The interpretation of the MM-GBSA results needs to be clarified. Specifically, the outcomes of the MM-GBSA analysis comparing the test and control compounds should be explained in detail.

**Response:** Utilizing the MD simulation trajectory, the binding free energy along with other contributing energy in form of MM-GBSA were determined for HAR+2-hydroxynaringenin. The results (Table 4) suggested that the maximum contribution to ΔG _bind _in the stability of the simulated complexes were due to ΔG _bind_Coulomb, ΔG _bind_vdW and ΔG _bind_Lipo, while, ΔG _bind_Covalent and ΔG _bind_SolvGB contributed to the instability of the corresponding complexes. The HAR+2-hydroxynaringenin complex showed significantly higher binding free energies. These results supported the potential of 2-hydroxynaringenin, showed the efficiency in binding to the selected protein and the ability to form stable protein-ligand complexes.

 Binding free energy components for the 1E3G+2-hydroxynaringenin and  1E3G+R18 calculated by MM-GBSA. Table is attached in the manuscript.
